# Using microbes as a key tool to unravel the mechanism of autophagy and the functions of the ATG proteins

**DOI:** 10.15698/mic2017.01.550

**Published:** 2016-12-30

**Authors:** Mario Mauthe, Fulvio Reggiori

**Affiliations:** 1 Department of Cell Biology, University Medical Center Groningen, University of Groningen, A. Deusinglaan 1, 9713 AV Groningen, The Netherlands.; 2 Department of Cell Biology, University Medical Center Utrecht, Center for Molecular Medicine, Heidelberglaan 100, 3584 CX Utrecht, The Netherlands.

**Keywords:** pathogens, picornavirus, unconventional function, virus, mechanisms, ATG13, FIP200

## Abstract

The study of microbe infections has always been a very effective approach to unveil and dissect cellular pathways. Autophagy is not an exception. Although some of the breakthrough discoveries in the field were obtained using yeast, pathogens have been and still are a great tool to discover and characterize new molecular and functional aspects of autophagy. Research on pathogens has helped to acquire knowledge about selective types of autophagy and the assembly of the autophagy machinery, i.e the autophagy-related (ATG) proteins, but also about alternative cellular roles of this pathway, such as secretion. Finally, microbes have also served to discover and characterize unconventional functions of the ATG proteins, which are uncoupled from their role in autophagy. In our recent study, we have taken advantage of viruses as a screening tool to determine the extent of the unconventional functions of the ATG proteome and characterize one of them.

## WHAT HAVE WE LEARNED ABOUT AUTOPHAGY BY STUDYING PATHOGEN INFECTIONS?

**Figure 1:**
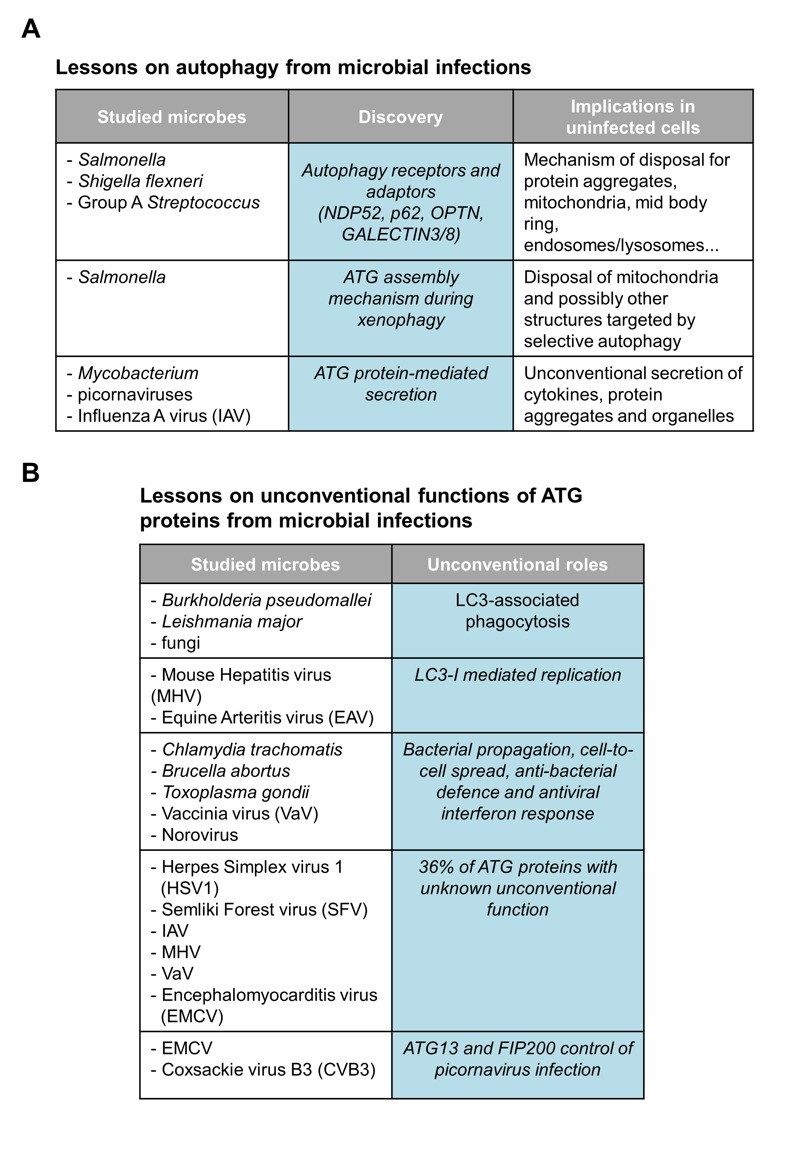
FIGURE 1: Tabular illustration resuming the studies mentioned in this commentary on microbes that have led to **(A)** the deciphering of autophagy-related processes and mechanisms, and **(B) **the identification of unconventional functions of the ATG proteins.

The hallmark of autophagy is the autophagosomes, double-membrane vesicles that originate from cistern structures called phagophores and that eventually fuse with lysosomes, where their sequestered cargo is turned over. For a long time, autophagy has been believed to be a non-selective, bulk degradation pathway within the cell, but recent evidences highlighted that determined stimuli trigger selective types of autophagy to target specific structures for lysosomal turnover 1. In mammalian cells, the investigation of bacterial infections has been an important source of knowledge for the mechanistic principles underlying selective types of autophagy (Fig. 1A). Selective degradation of pathogens by autophagy, also defined as xenophagy, was initially described for the intracellular elimination of *Group A Streptococcus *2. Another extensively studied model for xenophagy is the infection of *Salmonella*. After escaping into the cytosol from phagosomes, *Salmonella* becomes rapidly ubiquitinated and is sequestered into autophagosomes. These observations have prompted several groups to investigate the proteins that are responsible to target (ubiquitinated) bacteria, but also other pathogens into autophagosomes. These so-called autophagy receptors, which include nuclear dot protein 52 kDa (NDP52), p62 and OPTINEURIN (OPTN), have been identified and characterized by various laboratories 3,4,5. Although several autophagy receptors recognize the same bacterium, their function is not redundant but rather cooperative in restricting bacterial growth. The key feature of these autophagy receptors is that they can simultaneously bind ubiquitin and microtubule-associated protein 1 light chain 3 (LC3). Thereby, they effectively mediate the sequestration of ubiquitinated bacteria into autophagosomes. These principles of xenophagy have also been shown to be valid for other selective types of autophagy, which permit the disposal of protein aggregates, mitochondria or mid body ring (Fig. 1A) 1. A common factor that connects different selective forms of autophagy is the Tank-binding kinase 1 (TBK1). TBK1 can phosphorylate multiple autophagy receptors (e.g. OPTN, p62, NDP52) and thereby enhances their binding to LC3. In particular, phosphorylation of OPTN by TBK1 promotes selective sequestration of microbes as well as mitochondria 6. In the context of *Salmonella* infection, the laboratory of Felix Randow has identified another molecule, GALECTIN8, which is involved in xenophagy 7. This lectin does not bind directly to the invading bacteria but rather interacts with the glycans present in the lumen of host cell organelles that get exposed when microbes, such as *Salmonella,* disrupt the membrane of the bacterium-containing compartments. These membrane remnants are recognized by GALECTIN8, which in turn recruits NDP52 and thereby initiates autophagosome formation in close proximity of the invading bacterium 7. In addition to GALECTIN8, GALECTIN3 also localizes to disrupted membranes during *Shigella* infection when the bacteria escape into the cytosol from phagosomes 8. Interestingly, GALECTIN3 also detects membranes of damaged lysosomes and acts as a signal for autophagy-mediated turnover of these organelles (Fig. 1A) 9.

The study of microbes, in particular of bacteria, has also been valuable to acquire knowledge about the mechanism that leads to the assembly of the ATG machinery during selective types of autophagy (Fig. 1A). During bulk autophagy, the ATG machinery assembles by following a distinct sequential hierarchy to initiate the autophagosome formation 10. Kageyama and colleagues have revealed that upon induction of the formation of autophagosomes that target invading *Salmonella*, the functional groups composing the ATG machinery act in parallel axes, rather than sequentially 11*.* In particular, although LC3 recruitment and lipidation in close proximity of the bacteria requires the ATG12-ATG5-ATG16 complex, this localization is independent of ATG9 or phosphatidylinositol-3-phosphate (PI3P) generation. Nonetheless, components of the LC3 conjugation system as well as ATG9 and FIP200, a subunit of the ULK kinase complex, are all required for suppression of* Salmonella* growth because they are all necessary for the autophagosome biogenesis. In line with these observations, Kageyama and colleagues also observed that ATG9 associates to the invading bacteria prior to LC3. This model has been strengthened by a recent study showing that the formation of an autophagosome in close proximity of *Salmonella* is also modulated by activated TBK1. TBK1 is recruited to the ubiquitinated bacteria by different redundant mechanisms. This event is required for the localization of WIPI2, a PI3P-binding ATG protein, to the nascent autophagosome but not for the recruitment of LC3 12. Similar findings were reported for the selective degradation of mitochondria where components of the ULK complex and ATG9 are recruited to the mitochondria earlier than LC3 and in a manner that does not require autophagy receptors 13. These findings imply that the assembly mechanism of the ATG machinery (in particular LC3 recruitment) and the principles underlying autophagosome biogenesis initialization are different between selective and non-selective autophagy.

The study of bacteria and viruses has also helped in learning about alternative functions of autophagy (Fig. 1A). It has recently been shown that the release of *Mycobacterium *from amoebas in a non-lytic fashion is mediated by a so-called ejectosome. Although the ATG machinery is not required for the generation of the ejectosome, it is crucial to maintain plasma membrane integrity during this *Mycobacterium* exit from host cells 14. Autophagy-mediated secretion is not only important for bacteria release from host cells but also for the egression of specific viruses. Influenza virus is an example of a virus where optimal cell release depends on ATG proteins. Influenza M2 protein binds LC3 and thereby directs autophagosomes to the plasma membrane where they are required for the filamentous budding and the stability of the virions 15. Another example is the release of picornaviral particles. In addition to employ a lytic mechanism, the virions gain access to the extracellular milieu through a non-lytic release that depends on the ATG machinery and autophagosome-like vesicles 16,17. Importantly, autophagy-mediated secretion has now been shown to be important for the extracellular export of endogenous factors such as cytokines, protein aggregates and organelles during developmental processes 18.

## PATHOGEN INFECTIONS IN THE PIONEERING DISCOVERY OF UNCONVENTIONAL FUNCTIONS OF THE ATG PROTEINS

Although ATG proteins are principally known for their role in autophagy, the study of infections has been groundbreaking in revealing the existence of a variety of so-called unconventional functions of ATG proteins (Fig. 1B), where one or more ATG proteins are required for a process distinct from autophagy 19. The most well-known of these unconventional functions is probably the LC3-associated phagocytosis (LAP) 20. During LAP, the single membrane of phagosomes is modified to enhance lysosomal degradation of phagocytosed material, such as bacteria. This modification is characterized by the recruitment of LC3 through its covalent conjugation to the phosphatidylethanolamine present in the phagosomal membranes and it appears to require all ATG proteins except those that are part of the ULK kinase complex. LAP is triggered by infections by a variety of pathogens, including *Burkholderia pseudomallei*, *Leishmania major* and fungi, through the stimulation of pattern recognition receptors including specific Toll-like receptors 20,21. In addition to this role in innate immunity, LAP is also involved in processing and facilitating antigen loading onto major histocompatibility complex class II during infections contributing to adaptive immunity and plays a role in the clearance of dead cells by macrophages 22,23.

LC3 is also essential for the replication of viruses such as the mouse hepatitis virus (MHV) 24 and equine arteritis virus (EAV) 25. MHV and EAV induce the formation double-membrane vesicles (DMVs) in host cells, which act as replicative platforms. DMVs were initially believed to be autophagosomes because of their double-membrane morphology. However, it has been shown that these structures are still formed in absence of ATG5 and ATG7 even if they are positive for LC3 26. A study from our laboratory 24 revealed that in agreement with these observations, non-lipidated LC3 localizes to DMVs and that virus replication is inhibited by LC3 depletion. Interestingly, we also showed that MHV-induced DMVs are derived from EDEMosomes, endoplasmic reticulum-derived vesicles required for the regulated removal of two factors involved in the ER quality control, i.e. EDEM1 and OS-9, which are also positive for non-lipidated LC3.

A variety of other investigations about *Chlamydi trachomatis*, *Brucella abortus*, *Toxoplasma gondii*, vaccinia virus or norovirus have revealed additional unconventional functions of other ATG proteins in bacterial propagation, cell-to-cell spread, bacterial defense or antiviral interferon response 19.

## A VIRUS-BASED SCREENING APPROACH TO IDENTIFY UNCONVENTIONAL FUNCTIONS OF ATG PROTEINS

In one of our recent studies 27, we decided to exploit viral infections to identify novel unconventional functions of the ATG proteins. We performed an ATG proteome-specific siRNA screen and used the replication from 6 different viruses (herpes simplex virus 1, semliki forest virus, influenza A virus, mouse hepatitis virus, vaccinia virus and encephalomyocarditis virus) expressing luciferase as a read-out tool to score for the involvement of one or more ATG proteins in viral replication. Importantly, our siRNA library also took in consideration the redundancy of specific ATG genes by combining the siRNA that targeted them. Furthermore, we performed the screen in 2 different cell lines to avoid cell type-specific effects.

Overall, we found that 36% of the ATG proteins have a cell line-independent function in viral replication that is not connected to their role in autophagy (Fig. 1B). This number is even higher when cell type-specific events are taken in consideration. For the tested viruses, we did not find any *ATG* gene that is altering replication of all examined viruses and we did not observe autophagy being required in a cell line-independent manner for any of them.

To validate the outcome of the screen, we investigated in more detail two ATG proteins that are part of the ULK complex, ATG13 and FIP200, in the context of encephalomyocarditis virus (EMCV) infection. Depletion of ATG13 and FIP200 led to an increase in EMCV replication in the screen, whereas the other members of the ULK complex did not. In the follow-up experiments we could show that ATG13 and FIP200 are inhibiting the replication not only of EMCV, but also of other members of the picornavirus family, i.e. coxsackie virus B3 and A21, and enterovirus 71. Although the precise unconventional role of ATG13 and FIP200 in picornaviral infection remains to be elucidated, we showed that these two proteins suppress picornavirus infection by interfering with their replication and not by impairing the cell entry level (Fig. 1B).

Altogether, our study is another example within many showing how microbes can be very useful biological tools. It also confirms that they can be utilized for the identification of novel functions of the ATG proteins.
